# Effects of a traditionally prepared *Glycyrrhiza glabra* root-based beverage on female reproductive function in rats

**DOI:** 10.3389/fnut.2025.1702655

**Published:** 2025-10-29

**Authors:** Merve Kocaoğlu, Hacer Sinem Büyüknacar, Aslı Cevher, Gülçin Dağlıoğlu, Emine Kılıç Bağır, Fatma Peyman Ertuğ, Olcay Kıroğlu, Cemil Göçmen

**Affiliations:** ^1^Department of Pharmacology, Faculty of Pharmacy, Cukurova University, Adana, Türkiye; ^2^Department of Biochemistry, Faculty of Medicine, Cukurova University, Adana, Türkiye; ^3^Department of Patology, Faculty of Medicine, Cukurova University, Adana, Türkiye; ^4^Department of Medical Pharmacology, Faculty of Medicine, Cukurova University, Adana, Türkiye

**Keywords:** *Glycyrrhiza glabra*, FSH, LH, estrogen, uterine contractility, female reproductive function, estrous cycle

## Abstract

*Glycyrrhiza glabra* (licorice) is widely known for its traditional medicinal uses, including in the form of root-based beverages prepared and consumed in various regions. In Eastern and Southeastern Anatolia, such beverages are commonly consumed as part of daily dietary practices. Despite the widespread use of licorice root, data on the physiological effects of traditionally prepared licorice beverages on the female reproductive system are limited. This study aimed to evaluate the effects of substituting drinking water with a traditionally prepared *G. glabra* root-based beverage (30 mg/mL/day) for 7 days on female reproductive function in rats. Thirty-two adult female Wistar rats were divided into two main groups: Control and *G. glabra*; (licorice). Each group was further subdivided based on the estrous cycle into Proestrus/Estrus (P/E) and Diestrus/Metestrus (D/M) phases. Animals were housed in metabolic cages, and the experimental group received freshly prepared root-based licorice beverage instead of drinking water for 7 days. Vaginal smears were used to determine cycle phases. Serum levels of LH, FSH, and estrogen were measured using ELISA. Isolated uterus experiments were conducted to assess spontaneous and oxytocin-induced contractility (1000 mU/mL). Histopathological evaluations were performed on ovarian, liver, and kidney tissues. Compared to controls, the *G. glabra* group showed a significant increase (*p* < 0.05) in LH and FSH levels, while estrogen levels remained unchanged. Both spontaneous and oxytocin-induced uterine contractions exhibited significantly reduced amplitude and area under the curve (AUC) in the licorice group. Histological analysis revealed a decrease in the number of corpus luteum during the P/E phase, and an increase in primary and antral follicles during the D/M phase. No significant histopathological alterations were observed in the liver or kidney tissues. These findings suggest that traditional *Glycyrrhiza glabra* root-based beverages may influence female reproductive function in an estrous cycle-dependent manner, potentially affecting both hormonal regulation and uterine contractility.

## 1 Introduction

Since the emergence of human civilization, the use of plants in medicine has held significant importance. *Glycyrrhiza glabra* (licorice) is among these medicinal plants, with the utilization of its roots for therapeutic purposes tracing back to ancient times (1). The genus *Glycyrrhiza*, belonging to the *Leguminosae (Fabaceae)* family, comprises approximately 30 species distributed across various regions of the world, including *G. glabra*, *G. eurycarpa*, *G. aspera, G. inflata*, *G. uralensis*, and *G. korshinskyi* (1).

Native to Russia and China, this genus also grows in Mediterranean countries, Southeastern Europe, and parts of Asia. In Turkey, six *Glycyrrhiza* species have been identified, among which *Glycyrrhiza glabra* is the most widespread. It is a perennial herbaceous plant commonly found throughout Anatolia, typically reaching a height of 1–1.5 m (2, 3). *Glycyrrhiza glabra* (*G. glabra*) root is a traditional beverage made especially in the Eastern Anatolia and Southeastern regions of Turkey. The extract obtained by water extraction of *G. glabra* root is known as licorice and is consumed in large quantities as a daily beverage in our country, mostly in the Southeastern and Eastern Anatolia Regions (4).

From a nutritional perspective, licorice root is a source of amino acids, polysaccharides, simple sugars, proteins and mineral salts, including phosphorus, calcium, potassium, sodium, zinc, iron, selenium, magnesium, silicon, copper and manganese, as well as starch, pectin, sterols and resins (5). Additionally, studies have reported the presence of tannins, estrogens, phytosterols (such as stigmasterol and sitosterol), vitamins (B1, B2, B3, B5, E, and C), coumarins and glycosides in licorice root (5). Licorice contains approximately 3–18% of 18β-glycyrrhizin and its derivative 18β-glycyrrhizic acid (GA), which are considered its primary active constituents. The main bioactive compounds identified in licorice include glycyrrhizic acid, glycyrrhetic acid, isoangustone A, glabrene, glabridin, isoliquiritin, isoliquiritigenin, liquiritin, formononetin, and licochalcone A (6).

In central regions of Turkey, *Glycyrrhiza glabra* root extract is also used in the production of wine. In Italy, boiled *G. glabra* root is traditionally used for its laxative properties, whereas in Egypt, it is mixed with tea and administered for the relief of sore throat symptoms (7).

*Glycyrrhiza glabra* root is one of the oldest and most widely used herbal remedies in the world, and many of its traditional applications remain in practice today. *Glycyrrhiza glabra* has many pharmacological effects, including anti-inflammatory and antioxidant, antitussive and expectorant, antiulcer, antimicrobial, antiviral, anticarcinogenic, and antimutagenic effects, as well as dermatological, hepatoprotective, sedative, neuroprotective, and antidepressant properties (1). Recent evidence further supports these pharmacological effects, with Zafar et al. (8) providing crucial mechanistic insights into the modulatory role of licorice on steroid metabolism and related signaling pathways.

A review summarizing preclinical and clinical studies on the effects of licorice root on the reproductive system and sex hormones highlighted its potential estrogenic and antiandrogenic properties, emphasizing the possible therapeutic use of certain licorice extracts in alleviating menopausal symptoms and improving infertility (9). These effects are thought to be primarily mediated by its constituents such as glabrene and isoliquiritigenin. Glabrene, in particular, is widely used as a phytoestrogen in the treatment of menopausal symptoms (10), while isoliquiritigenin demonstrates potent estrogen-like activity (11). Powers and Setzer (12) reported that the stimulatory effects of glabrene were comparable to those of estradiol.

Some studies have shown that some flavonoids and chalcones derived from licorice root may inhibit enzymes involved in steroidogenesis or affect ovarian and uterine physiology by acting as partial estrogen receptor agonists (13, 14). However, findings regarding both beneficial and adverse effects on reproductive parameters are controversial.

Fertility represents a fundamental aspect of human life, reflecting not only its biological basis in reproduction but also its broader social, environmental and evolutionary implications (15). Despite the ongoing global population growth, an estimated one in six people worldwide will experience reproductive issues related to infertility during their lifetime, making infertility a major global health concern (16). The normal physiological regulation of female reproductive efficiency involves the hypothalamus secreting gonadotropin-releasing hormone (GnRH) in a pulsatile manner, which stimulates the pituitary gland through the hypothalamic–pituitary–gonadal axis to regulate the secretion of luteinizing hormone (LH) and follicle-stimulating hormone (FSH). In addition, the ovaries, with their enzymatic systems and steroidogenesis, produce sex steroid hormones such as estrogen and progesterone, while the uterus responds to these hormones to maintain reproductive function (17). However, the potential influence of traditional herbal preparations such as *Glycyrrhiza glabra* root-based beverages on these regulatory mechanisms has not been sufficiently investigated. Based on its phytochemical composition, we hypothesized that short-term consumption of *G. glabra* root-based beverage may modulate pituitary gonadotropin release, ovarian activity, and uterine contractility in a cycle-dependent manner.

Therefore, this study aimed to investigate the potential effects of traditionally prepared *Glycyrrhiza glabra* root-based beverage on uterine contractions and ovarian tissues of female rats in Proestrus/Estrus and Diestrus/Metestrus phases. Additionally, blood samples collected from the rats were analyzed for serum FSH, LH and estrogen levels using the ELISA method.

## 2 Materials and methods

### 2.1 Animal

Thirty-two adult female Wistar rats weighing 250–300 g were used in the experiments. The experimental animals were obtained from the Çukurova University Health Sciences Experimental Application and Research Center (SABIDAM). All animals were kept under standard laboratory conditions of 12 h light/12 h dark and humidity (40–60%) were controlled with air conditioning. The experimental animals were placed in metabolic cages to maintain normal living conditions and they received a standardized dose of a traditionally prepared *Glycyrrhiza glabra* root-based beverage.

The study was approved by the ethics committee of Çukurova University Animal Experiments Local Ethics Committee with the decision number 04-05-2023-3/8.

### 2.2 Experimental protocol

The study was conducted on two main experimental groups, each consisting of two subgroups with eight rats in each subgroup.

*Group 1-Control Group:*

Group 1-a: Proestrus/Estrus (P/E) phase *n* = 8.

Group 1-b: Diestrus/Metestrus (D/M) phase *n* = 8.

*Group 2-Glycyrrhiza glabra Root Group:*

Group 2-a: Proestrus/Estrus (P/E) phase *n* = 8.

Group 2-b: Diestrus/Metestrus (D/M) phase *n* = 8.

In the control group (Group 1), animals were provided with standard chow and regular drinking water. In the *Glycyrrhiza glabra* root group (30 mg/mL/day; Group 2), the animals received a fixed concentration of a traditionally prepared *Glycyrrhiza glabra* root-based beverage along with their standard diet. Rats consumed an average of 25.24 ± 1.70 mL of the beverage per day.

24-h water and/or *Glycyrrhiza glabra* consumption of rats kept in metabolic cages was measured. This protocol continued for 7 days. On the 8th day, the estrous cycle phases of the experimental animals were determined via vaginal smear analysis. Subsequently, the animals were anesthetized with 4–5% (v/v) sevoflurane, and blood samples were collected. Under deep anesthesia, the rats were then decapitated. The abdominal cavity was opened, and the uterus and ovaries were removed intact.

### 2.3 Preparation of a traditionally prepared *Glycyrrhiza glabra* root-based beverage

Root of *Glycyrrhiza glabra* was purchased from the local market in Adana, Turkey in May 2024; the specimen was identified by Doç. Dr. Serpil Demirci KAYIRAN (Department of Pharmaceutical Botanic, Cukurova University, Faculty of Pharmacy, Turkey). The licorice root-based beverage was freshly prepared daily in accordance with the method commonly used by the local population and in line with the dosage recommendations provided in the European Medicines Agency (EMA) pharmacopoeia (2013), Committee on Herbal Medicinal Products (HMPC): *Assessment report on Glycyrrhiza glabra L. and/or Glycyrrhiza inflata Bat. and/or Glycyrrhiza uralensis Fisch, radix, final. Doc. Ref.: EMEA/HMPC/571122/2010 Corr.1, 12 March 2013* (1995–2017). According to this monograph, 4.5 g of licorice root were infused in 150 mL of boiling water for 10 min. The infusion was then filtered to make it suitable for animal consumption. The prepared licorice root-based beverage was administered to the animals housed in metabolic cages over a period of 7 days (30 mg/mL/day).

### 2.4 Determination of estrous cycle stages via vaginal smear in rats

Due to the difficulty in distinguishing all stages of the estrous cycle during the experiments, closely related phases were grouped together and classified into two main stages: (P/E) and (D/M). According to this method, a vaginal smear was collected by gently inserting a cotton swab moistened with physiological saline into the vagina. The sample was then spread onto a microscope slide and stained by covering it with May-Grünwald eosin-methylene blue solution for 15 min. After rinsing, the slides were further stained with a diluted Giemsa solution for 30 min, followed by another rinse. Once dried, the slides were examined under a light microscope for evaluation, and the estrous cycle phases were identified accordingly.

### 2.5 Biochemical analyses

Following the completion of the 7-day experimental protocol, approximately 2 mL of blood was collected from each rat via intracardiac puncture under sevoflurane anesthesia using BD Vacutainer® SST™ II Advance yellow-capped gel tubes. The collected blood samples were centrifuged using a Nüve NF 1200 series centrifuge at 2000 rpm for 20 min to obtain serum. The serum samples were aliquoted in 0.5 mL portions into Microcult-brand Eppendorf tubes and stored at −20 °C. Prior to analysis, the samples from both the control and experimental groups were thawed at +4 °C and vortexed. Serum levels of FSH, LH, and estrogen were measured. The quantification of all three parameters was performed using enzyme-linked immunosorbent assay (ELISA), specifically based on the sandwich ELISA technique.

FSH levels (ELISA kit catalog no: 201-11-0183), LH levels (catalog no: 201-11-0180), and estrogen levels (catalog no: 201-11-0177) were measured using commercial kits supplied by SunRed Biotechnology. The analyses were conducted using a CHROMATE semi-automated micro-ELISA reader located at the Central Laboratory of the Faculty of Medicine, Çukurova University, following the manufacturer’s instructions. Results were expressed as follows: FSH in IU/L, LH in mIU/mL, and estrogen in ng/L.

### 2.6 *In vitro* isolated uterus experiments

Rats were anesthetized with 4–5% (v/v) sevoflurane and euthanized by decapitation. Subsequently, the abdominal cavity was opened, and the uterus tissue was excised for *in vitro* experiments. The tissue was isolated in a Petri dish containing Krebs solution (119 mM NaCl, 4.6 mM KCl, 1.5 mM CaCl_2_, 1.2 mM MgCl_2_, 15 mM NaHCO_3_, 1.2 mM NaHPO_4_, and 11 mM glucose). The uterus was then placed between two electrodes in a 5 mL organ bath in Krebs solution, maintained at 37 °C, and ventilated with 95% O_2_ and 5% CO_2_ under 1 g tone. Baseline spontaneous contractions were recorded for 60 min at the end of the 60-min incubation period. Then oxytocin (1000 mU/mL) was administered and phasic contraction occurred. These contractions observed for least 10 min. The bath was then washed with fresh Krebs solution to remove residual chemicals, and recordings continued until the amplitude of spontaneous contractions returned to pre-oxytocin baseline levels. Finally, to verify tissue viability, a contraction was induced with Krebs solution containing 100 mM KCl, marking the completion of the experiment. The same experimental protocol was applied across all groups. Tissue responses were recorded via isometric transducers connected to a data acquisition system (BIOPAC-MP36 Systems, Inc., Goleta, CA). All contractile responses were digitally recorded for subsequent analysis.

### 2.7 Histopathological evaluations

Routine follow-up procedures were applied to liver, kidney and ovarian tissues that were fixed in 10% buffered formalin. 5 micron thick H&E sections were taken from paraffin embedded tissues. The sections were examined under an Olympus BX46 light microscope.

### 2.8 Statistical analysis

The spontaneous/oxytocin contractile responses were determined by measuring mean maximum amplitude (g) and area under the curve (AUC; g.sec). The spontaneous and phasic contractions obtained were expressed as a percentage relative to the contraction induced by 100 mM KCl applied at the end of each experiment. Data were expressed as mean ± standard error. For the statistical evaluation of paired groups, the Student’s *t*-test was used. *p*-values smaller than 0.05 were considered statistically significant. Statistical analysis was performed with GraphPad Prism 5 software (San Diego, CA).

### 2.9 Drugs

Krebs solution and *Glycyrrhiza glabra* root-based drink were freshly prepared on a daily basis (30 mg/mL/day). Oxytocin was used at a concentration of 1000 mU/mL. For estrous cycle determination, May-Grünwald eosin-methylene blue solution (Merck) and Giemsa staining solution were utilized during the experiments.

## 3 Results

### 3.1 Microscopic images of estrous cycle phases

**Figure 1:**
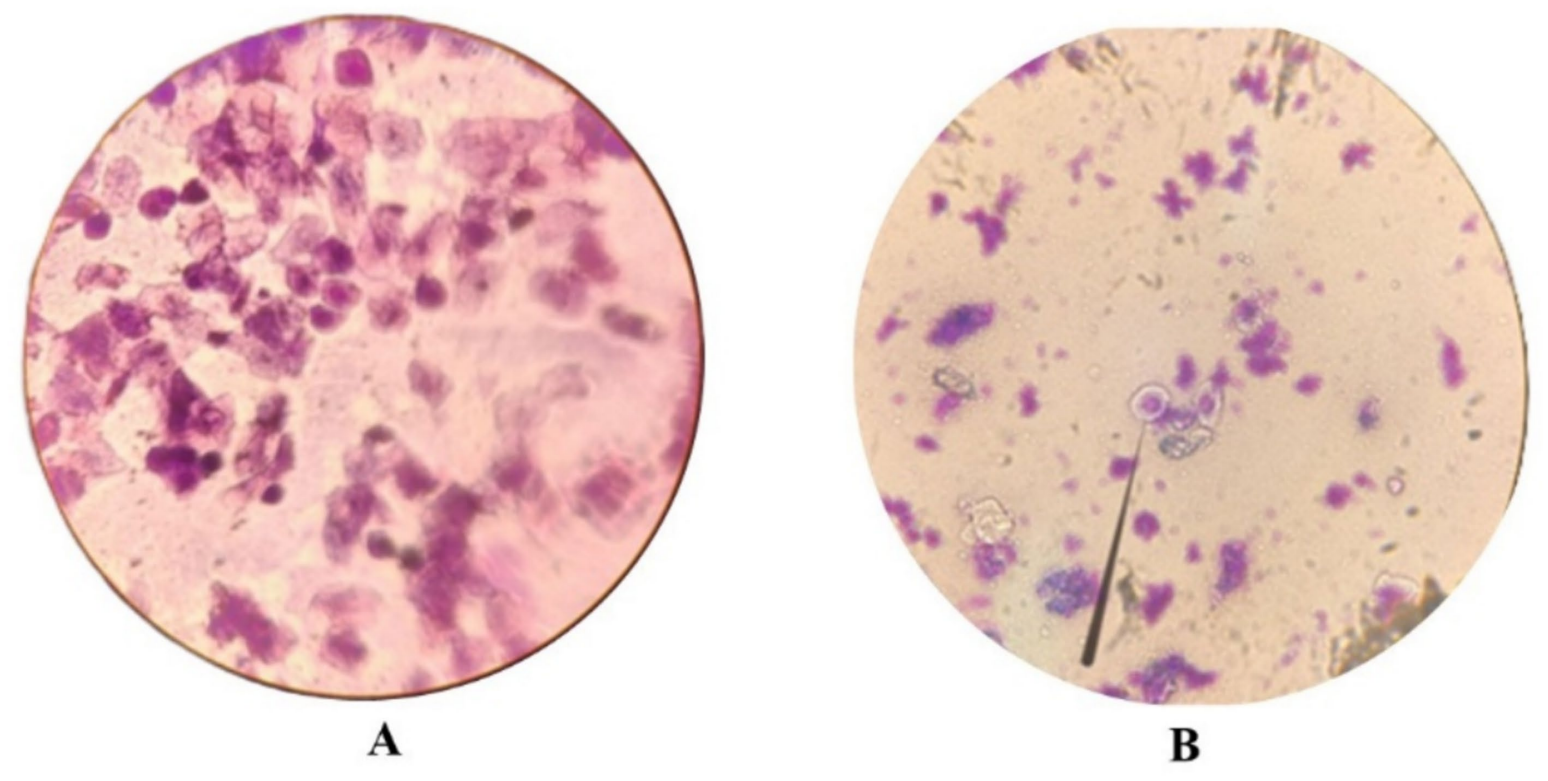
Microscopic images of vaginal smears showing the estrous cycle phases: (A) Proestrus/Estrus (P/E) and (B) Diestrus/Metestrus (D/M).

Vaginal smears were used to determine the phases of the estrous cycle, and due to the close similarity between certain stages, the phases were grouped into two main categories: Proestrus/Estrus (P/E) and Diestrus/Metestrus (D/M) (Figure 1).

### 3.2 Effect of *Glycyrrhiza glabra* root on estrogen levels

**Figure 2:**
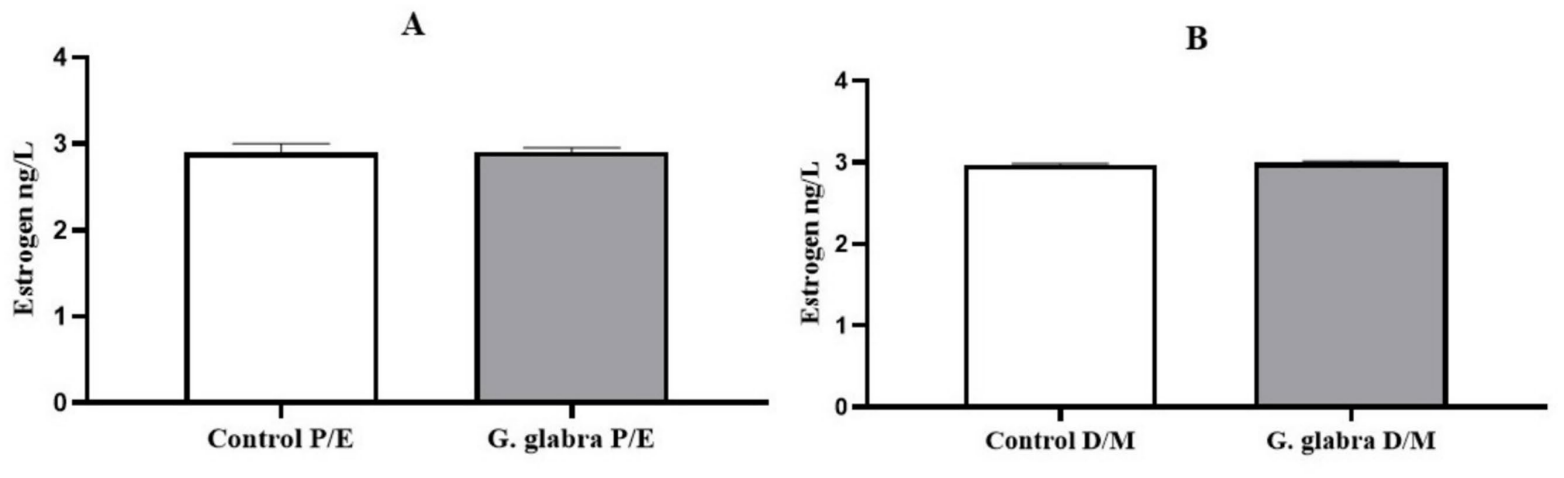
Measurement of estrogen levels (ng/L) in control and *G. glabra*-treated rats. **(A)** P/E, Proestrus/Estrus phase; **(B)** D/M, Diestrus/Metestrus phase (*n* = 8).

Estrogen levels were compared between the control group and the *G. glabra*-treated rats over a 7-day period, based on their estrous cycle stages. No statistically significant differences were observed between the control and *G. glabra*-treated groups in either the P/E or D/M phases (Figure 2).

### 3.3 Effect of *Glycyrrhiza glabra* root on LH levels

**Figure 3:**
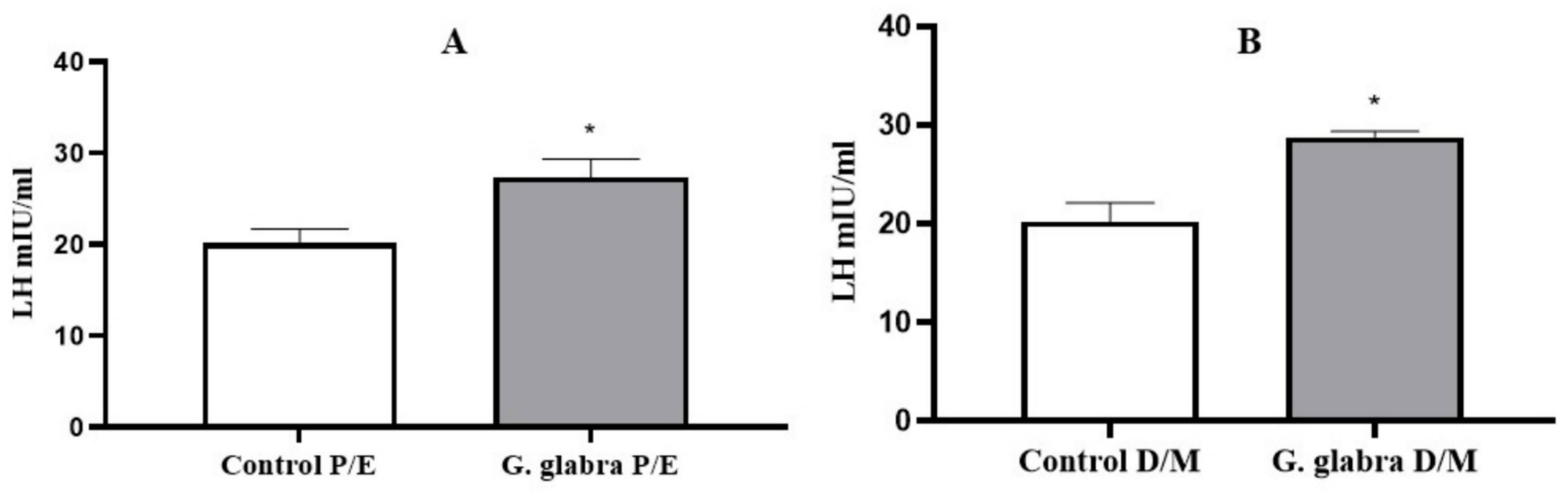
Measurement of LH levels (mIU/mL) in control and *G. glabra*-treated rats. **(A)** P/E, Proestrus/Estrus phase; **(B)** D/M, Diestrus/Metestrus phase. *p < 0.05 compared to the control group (*n* = 8).

LH levels were measured in both control and *G. glabra*-treated rats based on their estrous cycle stages. In both P/E and D/M phases, LH levels were significantly elevated in the *G. glabra*-treated groups compared to the control group (Figure 3, *p* < 0.05).

### 3.4 Effect of *Glycyrrhiza glabra* root on FSH levels

**Figure 4:**
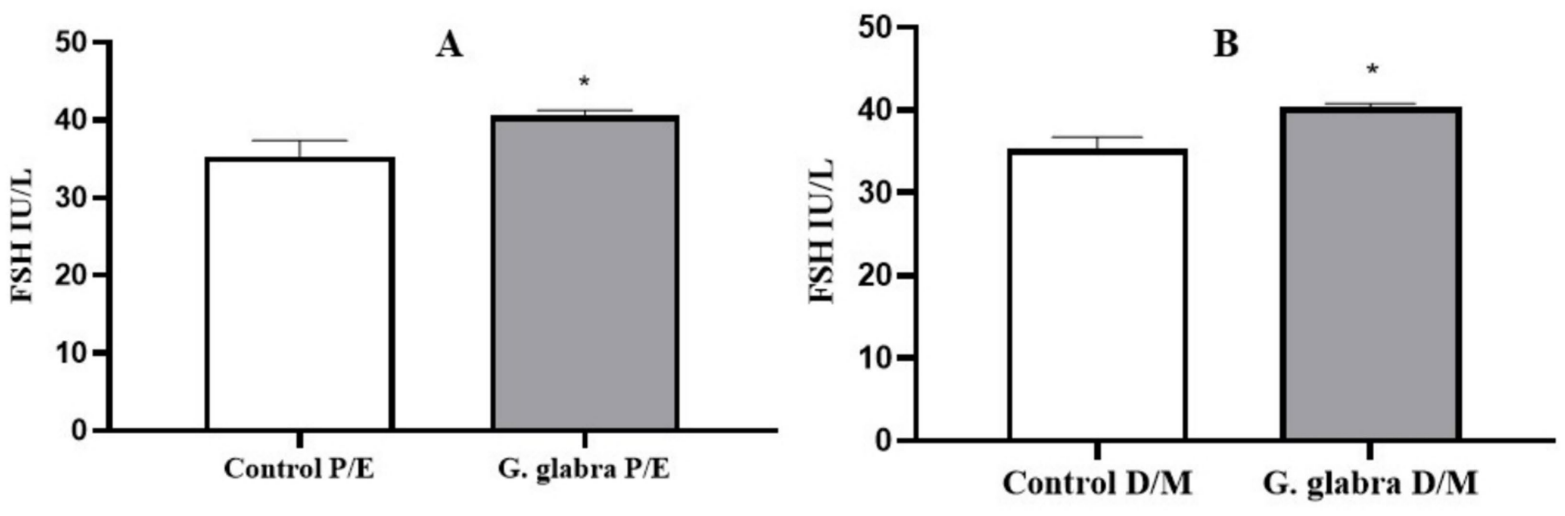
Measurement of FSH levels (IU/L) in control and *G. glabra*-treated rats. **(A)** P/E, Proestrus/Estrus phase; **(B)** D/M, Diestrus/Metestrus phase. *p < 0.05 compared to the control group (*n* = 8).

FSH levels were evaluated in control and *G. glabra*-treated rats based on their estrous cycle stages. In both the P/E and D/M phases, FSH levels were significantly increased in the G. glabra-treated groups compared to the control group (Figure 4, *p* < 0.05).

### 3.5 Effect of *Glycyrrhiza glabra* root on *in vitro* contractile responses

**Figure 5:**
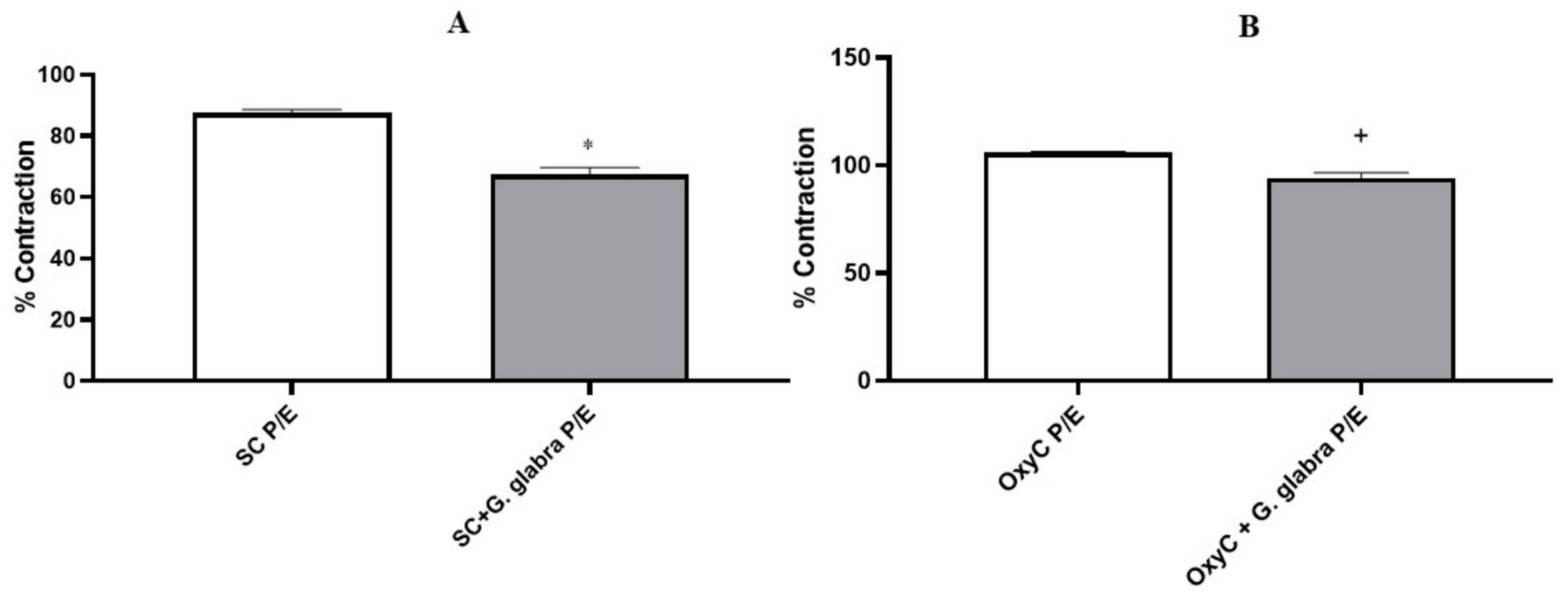
Percentage of contractile responses in control and *G. glabra*-treated rats, P/E: Proestrus/Estrus; **(A)** SC, spontaneous contractions; **(B)** OxyC, oxytocin-induced phasic contractions. *p < 0.05 compared to the spontaneous contractions. +*p* < 0.05 compared to the oxytocin-induced phasic contractions (*n* = 8).

**Figure 6:**
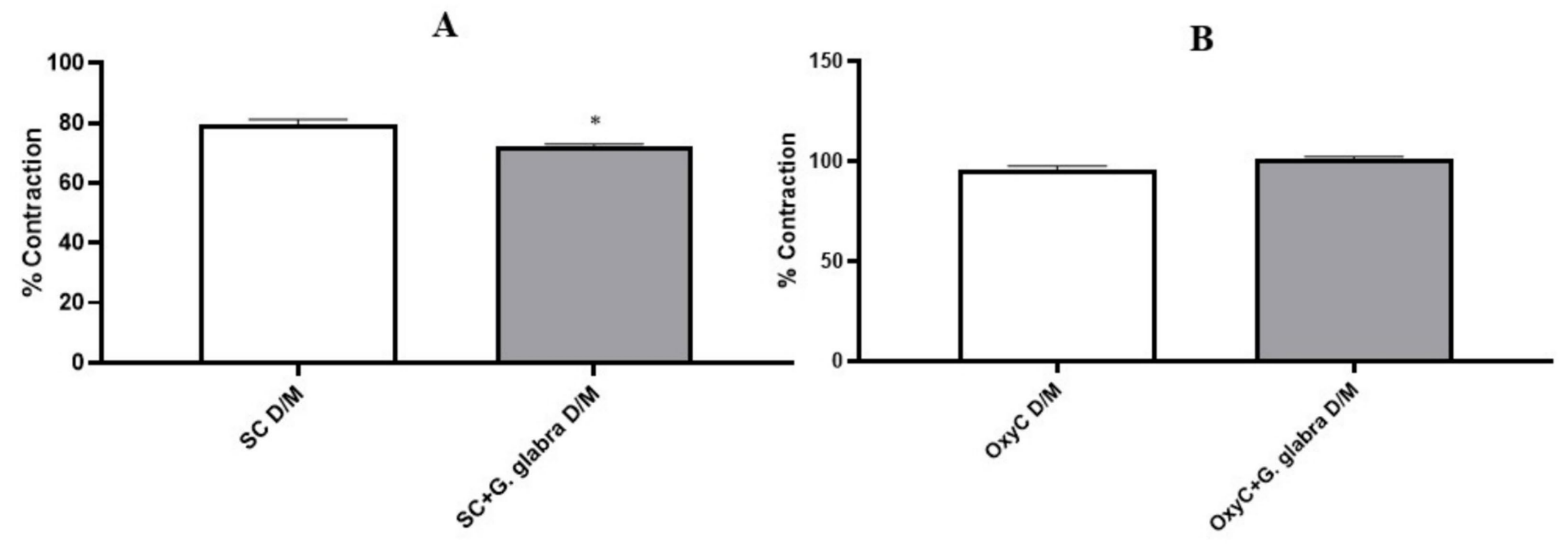
Percentage of contractile responses in control and *G. glabra*-treated rats, D/M: Diestrus/Metestrus; **(A)** SC, spontaneous contractions; **(B)** OxyC, oxytocin-induced phasic contractions. *p < 0.05 compared to the spontaneous contractions (*n* = 8).

In isolated uterine tissues, both spontaneous contractions and oxytocin-induced phasic contractions showed significantly reduced amplitude expressed as a percentage of KCl-induced contractions in the presence of licorice root during both the P/E and D/M phases (Figures 5, 6, *p* < 0.05).

**Figure 7:**
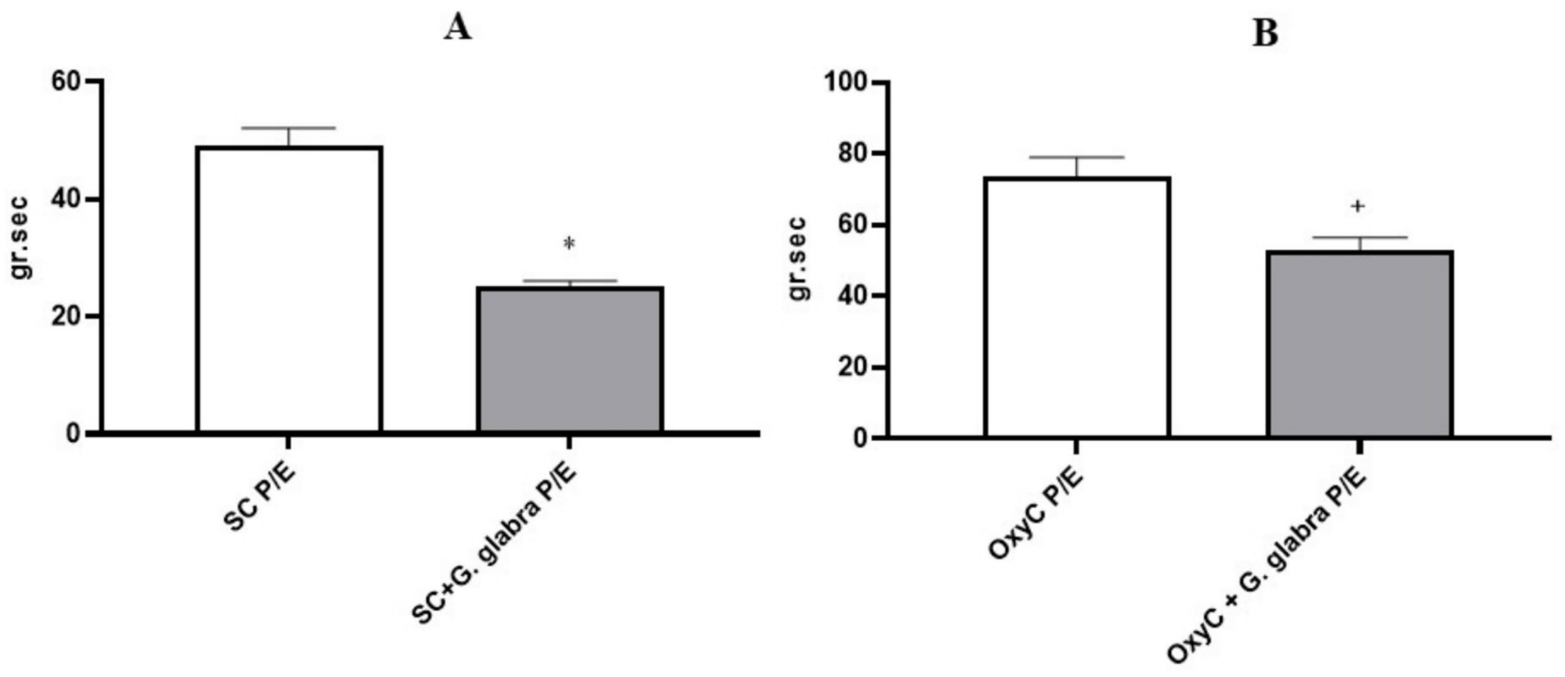
AUC of spontaneous and oxytocin-induced contractile responses in control and *G. glabra*–treated rats, P/E, Proestrus/Estrus; **(A)** SC, spontaneous contractions; **(B)** OxyC, oxytocin-induced phasic contractions. *p < 0.05 compared to the spontaneous contractions. +*p* < 0.05 compared to the oxytocin-induced phasic contractions (*n* = 8).

**Figure 8:**
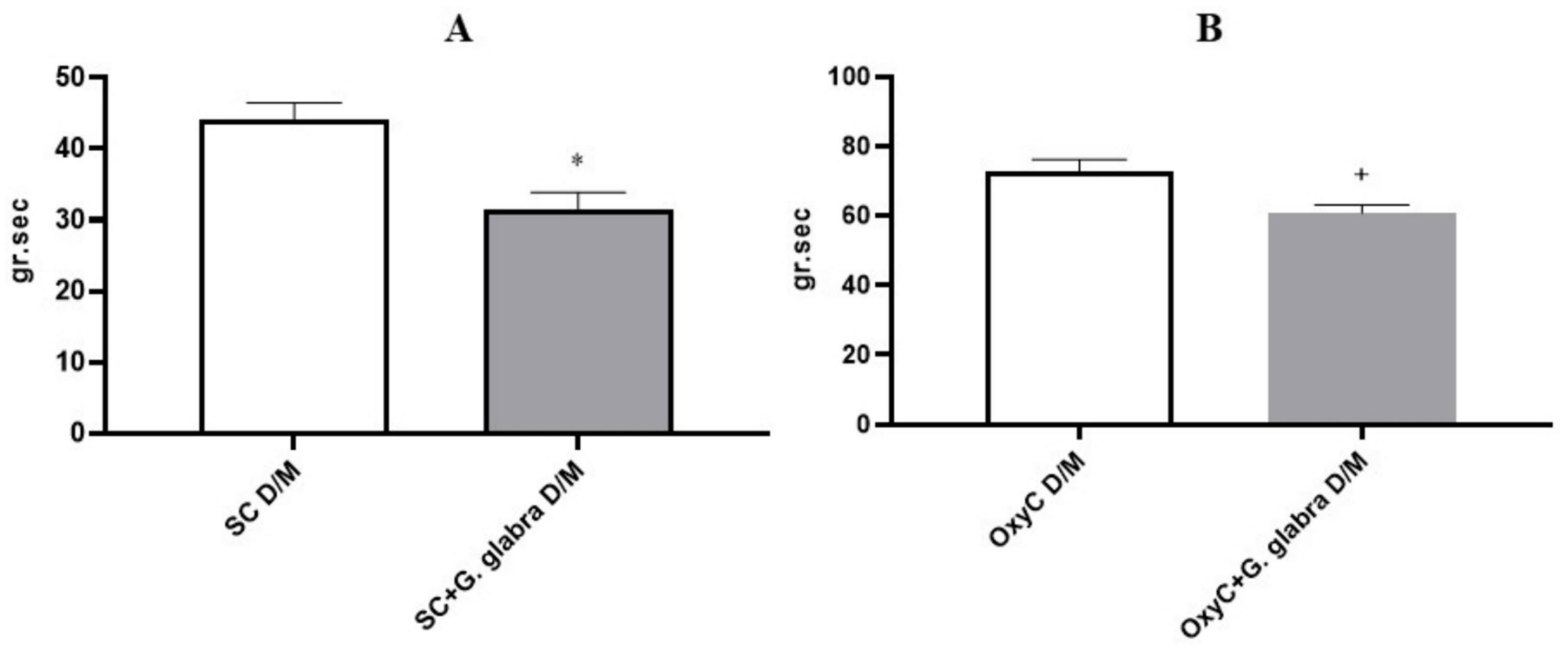
AUC of spontaneous and oxytocin-induced contractile responses in control and *G. glabra*-treated rats, D/M, Diestrus/Metestrus; **(A)** SC, spontaneous contractions; **(B)** OxyC, oxytocin-induced phasic contractions. *p < 0.05 compared to the spontaneous contractions. +*p* < 0.05 compared to the oxytocin-induced phasic contractions (*n* = 8).

The area under the curve (AUC; g.sec) of spontaneous contractions and oxytocin-induced contractile responses in isolated uterine tissues was significantly reduced in the presence of *Glycyrrhiza glabra* root during both the Proestrus/Estrus and Diestrus/Metestrus phases (Figures 7, 8, *p < 0.05*).

### 3.6 Histopathological evaluations

In both the control and *G. glabra*-treated rat groups, the numbers of antral follicles, primary follicles, and corpus luteum in the ovaries were evaluated. According to the results, a significant decrease in the number of corpus luteum was observed in the *G. glabra*-treated rats during the P/E phase compared to the control group. In contrast, the numbers of primary follicles and antral follicles were significantly increased in the *G. glabra-*treated rats during the D/M phase (Table 1).

**Table 1:** Numbers of primary follicles, antral follicles, and corpus luteum in rats during the P/E and D/M cycles in control and *G. glabra*-treated groups.

	PF	AF	CL
Control P/E	8.5 ± 0.9574	5.875 ± 0.9531	22.75 ± 2.056
*G. glabra* P/E	6.5 ± 0.2887	4 ± 0.6963	10.8^**+**^ ± 0.8
Control D/M	4.8 ± 1.2	4.429 ± 0.3689	16 ± 0.4082
*G. glabra* D/M	8.375^*^ ± 0.8851	6.5^*^ ± 0.3416	14.63 ± 0.6250

PF, Primary follicle; AF, Antral follicle; CL, Corpus Luteum. *p < 0.05 compared to the control D/M. +*p* < 0.05 compared to the control P/E.

In liver samples, mild sinusoidal dilatation, congestion, and, in some samples, mild portal inflammations were observed, but not significantly in both the control and *G. glabra* groups.

**Figure 9:**
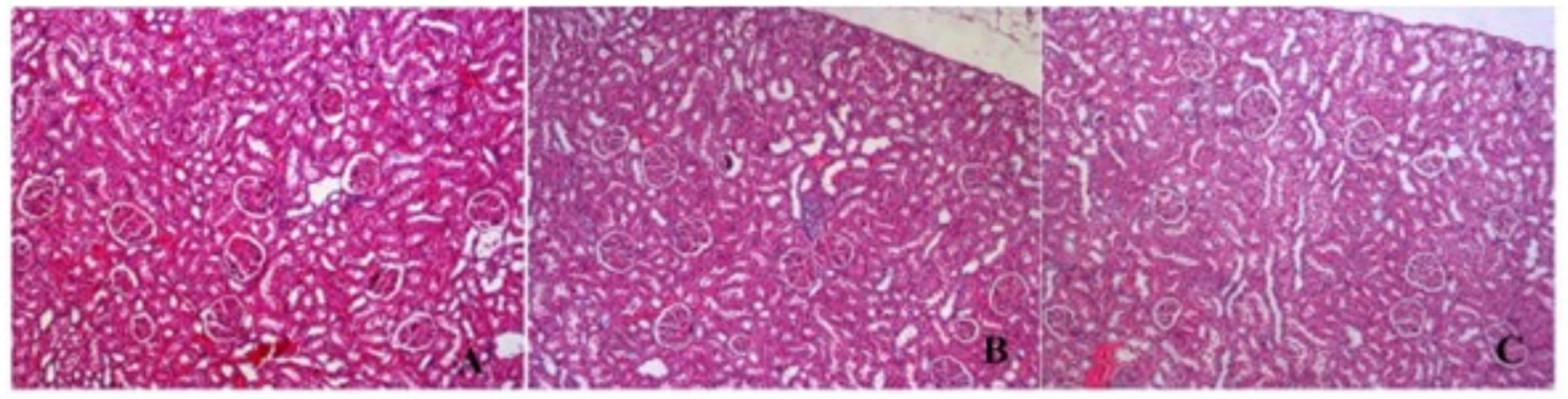
H&E section in kidneys samples congestion, mild interstitial inflammation and hyaline casts in some tubules. **(A)** Control (H&E X100) **(B)** P/E: Proestrus/Estrus phase, (H&E X100) **(C)** D/M: Diestrus/Metestrus phase. (H&E X100).

**Figure 10:**
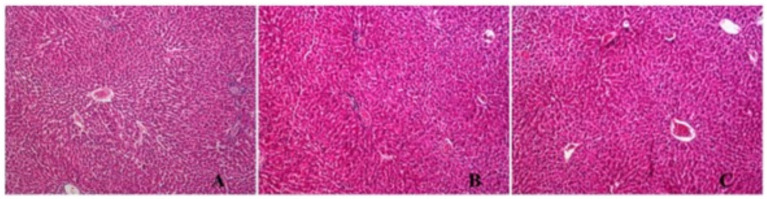
H&E section in liver samples mild sinusoidal dilatation, congestion and in some samples mild portal inflammation **(A)** Control (H&E X100), **(B)** P/E: Proestrus/Estrus phase (H&E X200), **(C)** D/M: Diestrus/Metestrus phase. (H&E X200).

In kidneys, no findings were observed except congestion, mild interstitial inflammation and hyaline casts in some tubules (Figures 9, 10).

## 4 Discussion

*Glycyrrhiza glabra,* commonly known as licorice root, is a perennial herbaceous plant from the family Fabaceae and is widely recognized for its pharmacologically active components. Rich in phytoestrogens, licorice root exhibits diverse therapeutic properties, including antidepressant, spasmolytic, laxative, antidiabetic, anti-ulcer, and anti-inflammatory effects (18, 19). Traditionally consumed as a herbal infusion in various cultures, licorice root has been suggested to influence hormonal pathways due to its phytoestrogenic constituents. Experimental and clinical studies have proposed that phytoestrogens can mimic or modulate estrogen receptor activity, potentially affecting reproductive health, menstrual cycle regulation, and fertility (20, 21).

In our study, a significant increase was observed in serum LH and FSH levels in rats administered licorice during both the P/E and D/M phases, while no significant change was found in estrogen (E2) levels. Despite the increase in gonadotropins, the lack of a significant change in E2 suggests that alternative feedback mechanisms may be involved in the hypothalamic–pituitary-ovarian axis.

Tamir and colleagues (21, 22) initially demonstrated that certain components and derivatives of licorice root, such as glabrene, isoliquiritigenin and glabridin act like phytoestrogens that bind to human estrogen receptors in various estrogen-responsive tissues. Glabridin has also been reported as a phytoestrogen that binds to the human estrogen receptor and stimulates creatine kinase activity in the rat uterus (22). These phytoestrogenic effects may theoretically influence hypothalamic–pituitary feedback loops. Therefore, licorice root may be used to improve female reproductive function and to treat female reproductive system disorders due to the presence of beneficial phytoestrogens and flavonoids. However, despite the observed increase in gonadotropins in our study, no significant change was detected in E2 levels. This suggests that the rise in LH/FSH may be attributed to alternative mechanisms such as increased pituitary sensitivity or enzymatic modulation in steroid biosynthesis.

In a study conducted by Mahalingam et al. (20), the licorice-derived chalcone isoliquiritigenin was shown to exert an inhibitory effect on antral follicle growth and steroidogenesis in *in vitro* mouse follicle culture studies, altering estradiol, testosterone, and progesterone levels. This finding supports the possibility that licorice components may directly affect ovarian steroidogenic enzymes (e.g., CYP17A1, aromatase, HSD17B1, STAR), potentially leading to a mismatch between elevated gonadotropin levels and ovarian response.

Due to its high content of phytoestrogenic compounds, one study has also demonstrated its potential therapeutic benefits in the treatment of estrogen-dependent conditions such as breast cancer, endometriosis, polycystic ovary syndrome (PCOS), and premature ovarian failure (POF) (23). A study shown that *Glycyrrhiza glabra* root inhibits two key enzymes-3β-hydroxysteroid dehydrogenase (3HSD) and 17β-hydroxysteroid dehydrogenase (17HSD)-while stimulating aromatase activity. It also affects the activity of 5α- and 5β-reductase enzymes, which are involved in the synthesis and metabolism of androgens and estrogens (24).

Due to the presence of phytoestrogens with aromatase-inducing and 17HSD-inhibiting activity, licorice root can be used as a potential therapeutic agent in the treatment of women with PCOS by reducing testosterone synthesis (25). Additionally, a study on mice with PCOS showed that *G. glabra* root extract improved oocyte maturation, embryonic development, and ovarian morphology in a dose-dependent manner compared to the control group (26).

In a study conducted by Yang et al. (27), administration of *Glycyrrhiza glabra* (0.3 g/kg) to six-week-old female Sprague Dawley rats with letrozole-induced PCOS for 2 weeks resulted in increased serum FSH levels, decreased LH: FSH ratio, and significant improvements in ovarian morphological parameters. This suggests that licorice components may possess modulatory effects on the gonadotropin-steroid axis. The findings of our study suggest that short-term traditional licorice intake may increase pituitary gonadotropin release in the early phase while having a limited effect on ovarian steroid response; this may raise the hypothesis that longer durations or different extract compositions may significantly alter follicular dynamics and steroidogenesis.

A 2020 study reported that licorice root decreased estrogen levels and increased FSH levels in ovariectomized rats. Licorice root has been observed to reduce depression, increase serum E2 and FSH concentrations for the endocrine system, and improve menopausal syndrome through multiple mechanisms by affecting the neuroendocrine-immune network (28). A study by Kim and Park (29) reported that isoflavones may affect sexual development and disrupt the estrous cycle as well as the functions of the ovaries, hypothalamus and pituitary glands.

In our study, histological examination of the ovaries, including corpus luteum and oocytes, was performed in all groups. Histopathological analysis revealed a marked decrease in the number of corpus luteum (CL) in the licorice group during the P/E phase, whereas an increase was observed in the number of primary and antral follicles during the D/M phase.

Shamsi et al. (26) recently evaluated the effects of licorice root extract on oocyte maturation, ovarian morphology and embryo development in female rats with PCOS, and demonstrated that high-dose oral supplementation of licorice (0.1–0.15 g/kg) resulted in significant improvements in oocyte morphology, evidenced by fewer cystic and atretic follicles and a greater number of healthy follicles compared to the PCOS group. Furthermore, Mahalingam et al. (20) show that isoliquiritigenin inhibited antral follicle growth and reduced the gene expression of steroidogenesis are consistent with our observed decrease in corpus luteum and the increase in gonadotropins, suggesting possible ovary-targeted effects.

The regulatory effects of licorice on uterine function are noted in classical Chinese texts such as Yi Zong Jin Jian, and in modern practice the licorice-containing formula Shakuyaku-kanzo-to (Shaoyao-Gancao-Tang) has been used to manage dysmenorrhea. Clinical and experimental studies have reported that Shakuyaku-kanzo-to and *Glycyrrhiza glabra* extracts can alleviate uterine spasms and improve dysmenorrhea symptoms (30, 31).

In a study conducted by Shi et al. (14) on mouse and rat uterine tissue, it was demonstrated *in vitro* that isoliquiritigenin inhibited both spontaneous contractions and oxytocin (1 mU/mL)-induced contractions in isolated rat uteri in a concentration-dependent manner. These effects are suggested to occur via mechanisms involving Ca^2+^ channels, COX, and NO pathways, indicating potential benefits in dysmenorrhea and various pain syndromes.

A study in rat jejunum demonstrated that a Shaoyao–Gancao decoction of *Glycyrrhiza* roots and rhizomes and the dried root slice of *Paeonia lactifora* Pall caused a significant reduction in spontaneous and induced contractions (32) In addition, licorice root has been reported to have a smooth muscle relaxant effect on carbachol-induced contractions in the mouse small intestine (33). Furthermore, a study found that licorice extract significantly inhibited both spontaneous phasic contractions and oxytocin-induced contractions in isolated uterine tissue from mice treated with stilbestrol. This finding suggests that licorice extract may exert a spasmolytic effect on isolated uterine tissue from non-pregnant mice (13).

In our study, unlike previous research, the effect of licorice root on uterine contractions was evaluated according to the estrous cycle phases in rats. In all phases, both the amplitude and the area under the curve (AUC) of spontaneous and oxytocin-induced contractions were significantly reduced in licorice-treated rats. This finding is consistent with other studies and suggests that licorice root may be effective in the treatment of dysmenorrhea.

In liver samples from the control and *G. glabra* groups, mild sinusoidal dilatation, congestion, and mild portal inflammation in some samples were observed, but this was not significant. In the kidneys, no findings were observed other than congestion, mild interstitial inflammation, and hyaline casts in some tubules. Consequently, upon histopathological examination of liver and kidney tissues, no significant differences were observed in licorice-treated liver and kidney tissue samples compared to control. However, glycyrrhizin, the main saponin component of licorice, and its metabolites have previously been associated with mineralocorticoid-like effects and electrolyte imbalances, indicating a potential toxicity profile (34). Therefore, close monitoring of hepatic and renal parameters is essential in studies involving long-term or high-dose licorice consumption.

## 5 Conclusion

In conclusion, traditionally prepared *Glycyrrhiza glabra* root-based beverage was shown to have a stimulatory effect on LH and FSH levels, which may be associated with ovulation, and to reduce both the amplitude and AUC of uterine contractile responses, suggesting a modulatory effect on uterine tone. The observed increase in the number of primary and antral follicles in licorice-treated groups may indicate an improvement in follicular reserve and a potential enhancement in the likelihood of conception. Likewise, the reduction in uterine activity and contraction duration may help lower the risk of miscarriage, thereby potentially supporting the maintenance of pregnancy. Overall, our findings suggest that *G. glabra* root-based drink may exert beneficial effects on the female reproductive system. However, these effects appear to vary depending on the stage of the estrous cycle.

## Data Availability

The original contributions presented in the study are included in the article/supplementary material, further inquiries can be directed to the corresponding author.
